# Hemodynamic Changes in the Right Ventricle Induced by Variations of Cardiac Output: A Possible Mechanism for Arrhythmia Occurrence in the Outflow Tract

**DOI:** 10.1038/s41598-018-36614-7

**Published:** 2019-01-14

**Authors:** Utku Gülan, Ardan Muammer Saguner, Deniz Akdis, Alexander Gotschy, Felix C. Tanner, Sebastian Kozerke, Robert Manka, Corinna Brunckhorst, Markus Holzner, Firat Duru

**Affiliations:** 1 ETH Zurich, Institute of Environmental Engineering, Zurich, 8093 Switzerland; 2 University Heart Center, Department of Cardiology, Zurich, 8091 Switzerland; 3 Center for Integrative Human Physiology, University of Zurich, Zurich, 8091 Switzerland; 4 Institute for Biomedical Engineering, University and ETH Zurich, Zurich, 8092 Switzerland; 5 Institute of Diagnostic and Interventional Radiology, University and ETH Zurich, Zurich, 8092 Switzerland

## Abstract

The rationale of this paper is to investigate right ventricular (RV) hemodynamics in relation to changes in cardiac output, and in particular to study exercise-induced stresses at the RV outflow tract (RVOT), which is a common site of ventricular arrhythmias in the athlete’s heart. We hypothesize that the thin-walled RVOT is exposed to high wall shear stresses (WSS) during physiological states associated with high cardiac output such as exercise, and therefore, may be particularly prone to substrate formation leading to ventricular tachyarrhythmias. 3D Particle Tracking Velocimetry (3D-PTV), an optical imaging method, has been performed in a novel anatomically accurate compliant silicone right heart model derived from a high resolution MRI heart scan of a healthy male proband. RV and RVOT flow patterns at resting conditions were obtained from two healthy athletic male proband’s hearts and two patients with arrhythmogenic right ventricular cardiomyopathy/dysplasia (ARVC/D) via phase contrast magnetic resonance imaging (PC-MRI). The healthy case was used as a reference for validating the *in vitro* flow patterns of the silicone model, while the diseased cases were used to generalize our findings and investigate possible changes in hemodynamic stresses with RV morphological remodelling. Our results showed that both healthy and diseased geometries consistently displayed an increased WSS in the RVOT relative to the rest of the RV. We found that increases in cardiac output may lead to increases of mean kinetic energy (MKE), laminar viscous dissipation and WSS at the RVOT. Furthermore, higher peak WSS magnitudes were found for the diseased cases. The identified high WSS regions may correlate with the common site of RVOT ventricular tachycardia in athletes and patients with ARVC/D. Our results imply that exercise, as well as anatomical and functional remodeling might alter RV wall shear stress both in magnitude and spatial distribution, leading to increased hemodynamic stresses in the RVOT.

## Introduction

Regular physical exercise reduces cardiovascular morbidity and stimulates a number of beneficial physiological changes in the human body, i.e. it improves insulin sensitivity, increases serum high density lipoprotein, coronary flow reserve and myocardial capillary density, and reduces the progression of atherosclerosis^1–3^. During exercise, cardiac workload, oxygen uptake and carbon dioxide output increase, which go along with an increase in cardiac output^4^. Hence, exercise may cause a distinctive stress on the cardiopulmonary circulation^4^. Most of the previously reported studies on endurance exercise have focused on the left heart^5,6^. However, it has been shown that endurance exercise predominantly affects the right ventricle (RV)^7^. Therefore, RV dimensions and function are clinically important parameters, which may provide an insight into exercise-induced alterations of myocardial function^8,9^.

Recent data suggests that regional structural alterations in the RV may not only be found in patients with ARVC/D, but also occur in apparently healthy endurance athletes in the absence of desmosomal mutations^10^. The extent of these alterations seems to be associated with the type, intensity and duration of exercise^11,12^. Scharhag *et al*. suggested that endurance training induces RV hypertrophy representing a physiologic adaptation^13^. Moreover, long-term endurance exercise training may cause dilatation of the RV and right atrium, RV diastolic dysfunction, and cardiac fibrosis, which may provide a substrate for right-sided ventricular arrhythmias, RVOT ventricular tachycardia in particular, and increase cardiovascular risk^3,14^. Furthermore, it has been demonstrated that the development of an arrhythmogenic substrate in the RV in athletes heavily relies on the intensity of physical activity^12,15,16^. Interestingly, regular intense endurance exercise may promote a differential segmentary remodeling between the RV base and RV apex, potentially leading to differences in wall stress at rest^15^.

Fluid mechanics parameters such as mean kinetic energy (MKE), turbulent kinetic energy (TKE) and wall shear stress (WSS) in the cardiovascular system have been proposed as potential markers for predicting the development of different cardiovascular pathologies. Fredriksson *et al*.^17^ suggested high TKE levels for the complications seen in Tetralogy of Fallot (ToF). Gulan *et al*.^18^ proposed a novel approach, which relies on TKE and dissipation of MKE for the assessment of aortic valve stenosis severity. Zajac *et al*.^19^ used TKE for determining diastolic dysfunction in the left ventricle of patients with dilated cardiomyopathy. Similarly, Dyverfeldt *et al*.^20^ utilized total kinetic energy levels to estimate the severity of mitral regurgitation. Binter *et al*.^21^ showed that TKE may provide complementary information to echocardiography, assisting to distinguish within the heterogeneous population of patients with moderate to severe aortic stenosis. Recently, Gulan *et al*.^22^ showed that vortex rings develop in the right atrium correlating with WSS, which may help to understand the formation of right atrial thrombus. It is also known that low WSS may lead to intimal wall thickening and hence promotes atherogenesis^23^.

RVOT tachycardia can be a primary electrical disease in the absence of structural heart disease^24,25^ but can also occur in athletes with subtle structural heart disease and patients with arrhythmogenic right ventricular cardiomyopathy/dysplasia (ARVC/D)^14,26^. It has been shown that exercise may provoke RVOT tachycardia^27^, yet the mechanisms for these observations have not been fully understood. The human RV has a complex geometry, which introduces challenges for echocardiographic analysis^9,13^. With the improvements in magnetic resonance imaging (MRI) on space and time resolved kinetic energy measurements, it is now possible to investigate flow patterns in the RV^28,29^. To the best of our knowledge, the effects of exercise training on blood flow patterns and associated shear stresses in the right heart have not yet been studied. We hypothesize that the RVOT free wall is exposed to high WSS during physiological states associated with high cardiac output, such as exercise, and therefore this region of the RV may be particularly prone to substrate formation leading to ventricular tachyarrhythmias. Since it is challenging to study cardiac output effects *in vivo*, we performed 3D-PTV flow measurements in an anatomically accurate compliant silicone right heart model. The results are validated and complemented by *in vivo* flow MRI measurements for two healthy and two pathological (ARVC/D) geometries.

## Results

### Hemodynamics in the healthy RV at resting condition

**Figure 1:**
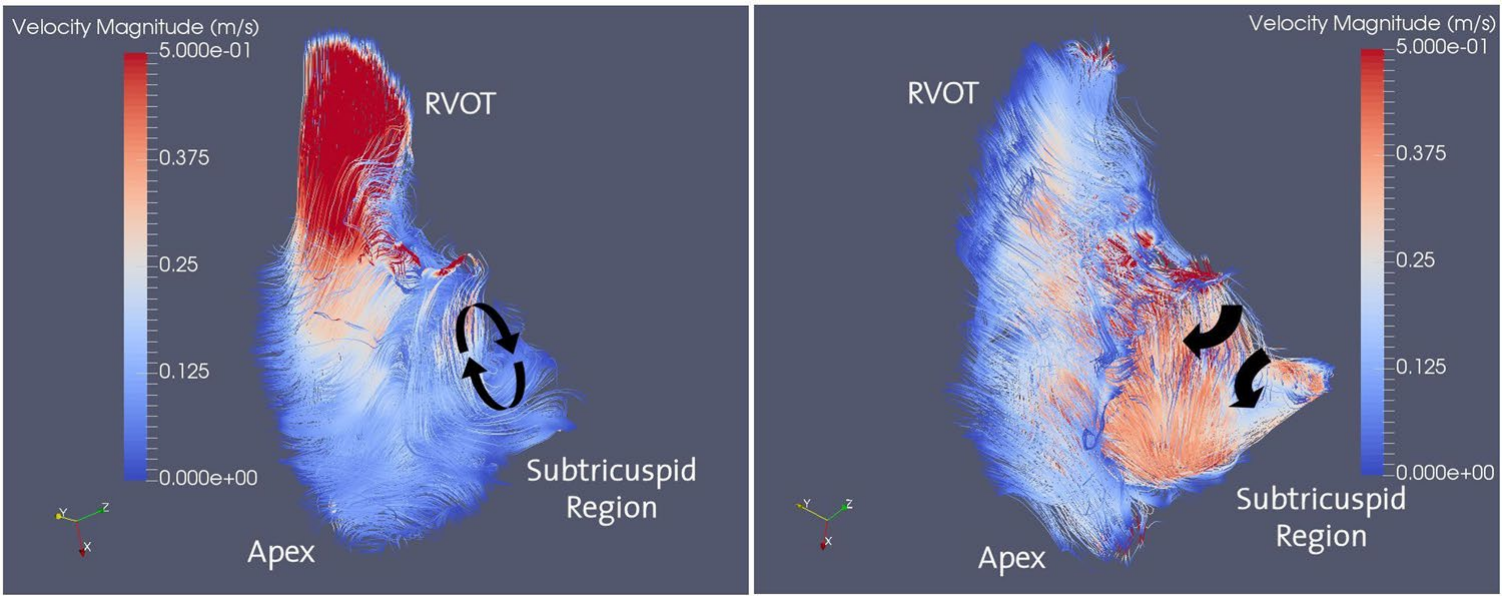
Streamlines color coded with velocity magnitude along the RV at peak systolic phase (left) and mid diastolic phase (right) obtained using *in vivo* PC-MRI in a healthy athletic male proband at resting conditions. Red color corresponds to high velocity and the blue color represents slow velocity regions. The PC-MRI data of the healthy male volunteer is used for qualitative representation of the blood flow and as a reference for validating the *in vitro* flow patterns of the silicone model (see Methods section). Recirculation regions at ventricular filling are marked with arrows.

This section focuses on *in vivo* blood flow characteristics at resting conditions. A qualitative representation of the *in vivo* blood flow patterns in the RV at peak systole and mid diastolic phases are shown in Fig. 1.

In the peak systolic phase (left), high velocity regions develop in the vicinity of the RVOT, particularly at the RVOT free wall. The RV forwards the ventricular blood volume into the pulmonary artery via contraction when the tricuspid valve closes and the pulmonary valve opens. Local recirculation regions arise distal to the RVOT, i.e. in the proximity of the subtricuspid region. In the ventricular filling phase (right), high velocity regions develop in the vicinity of the subtricuspid region as the tricuspid valve opens and the pulmonary valve closes. The RV expands as the atrial blood volume fills the RV. At the RVOT, retrograde flow zones arise with slow velocity. It is noted that recirculation regions develop in the proximity of the tricuspid valve, which are associated with a diastolic vortex present in that region. A major portion of the RV flow is directed towards the RVOT and a small portion of the volume is trapped in the recirculation zone.

**Figure 2:**
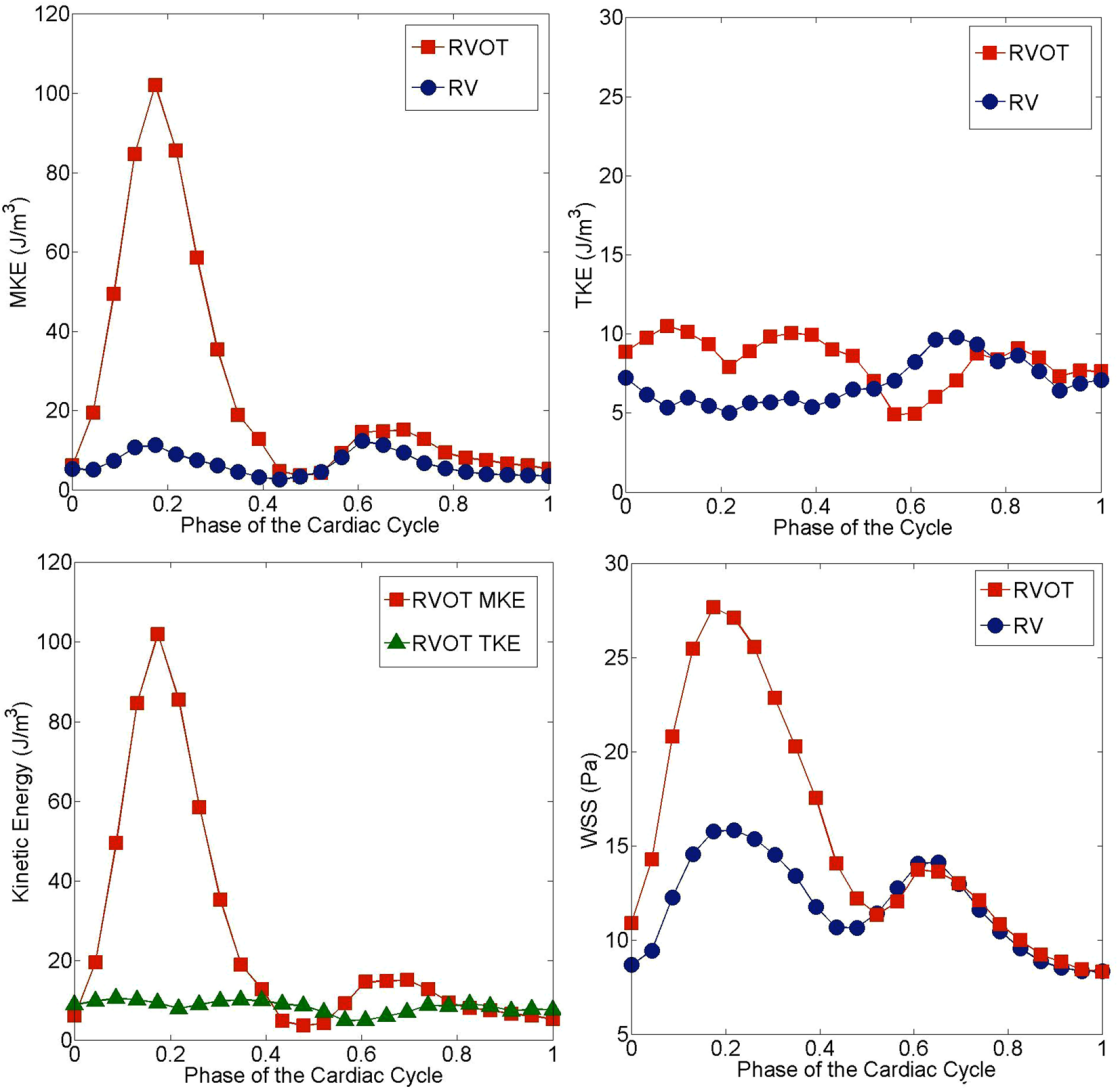
Time evolution of MKE averaged over the RVOT and RV (top left), temporal variation of TKE averaged over the RVOT and RV (top right), comparison of the time evolution of kinetic energies at the RVOT (bottom left) and time evolution of WSS averaged over the RVOT and RV (bottom right) obtained using *in vivo* PC-MRI in a healthy athletic male proband at resting conditions. Red squares correspond to the RVOT, blue circles represents the entire RV and green triangles correspond to the RVOT TKE.

The time variation of MKE averaged over the RVOT and RV (Fig. 2, top left) shows that there is a difference in MKE levels between the RVOT and RV at peak systole. Given that high velocity regions develop at the RVOT during ventricular systole (Fig. 1, left), it is expected that high MKE zones arise at the RVOT. An order of magnitude increase in RVOT MKE may result in strong spatial gradients of velocity and hence elevated energy dissipation and shear stresses at the RVOT, which we investigate in detail below. On the other hand, the time evolution of TKE averaged over the RVOT and RV shows that both RVOT and RV TKE values are in the same range (Fig. 2, top right). In Fig. 2 (bottom left), we show that RVOT MKE is considerably higher than RVOT TKE, which implies that there is some turbulence during the systolic phase, but it is not particularly strong. Finally, we note that WSS at the RVOT during peak systole is twice as high as the WSS averaged over the entire RV including the RVOT (Fig. 2, bottom right). Conversely, in the diastolic phase, WSS levels become similar for both RVOT and RV.

### Effects of changes in cardiac output on *in vitro* flow characteristics

**Figure 3:**
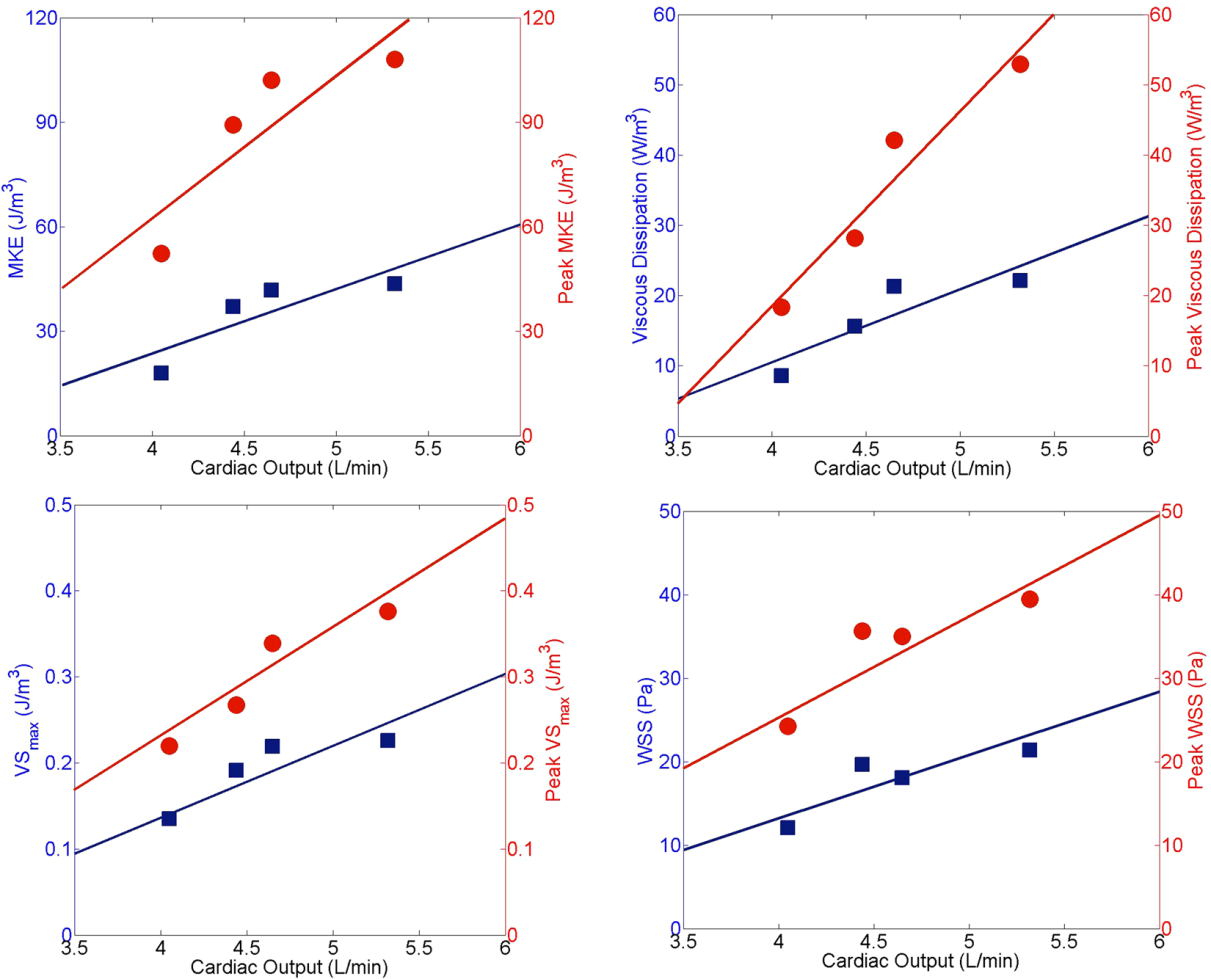
MKE and peak MKE averaged over the RVOT for varying cardiac output levels (top left). Laminar viscous dissipation and peak laminar viscous dissipation averaged over the RVOT for varying cardiac output levels (top right). Maximum viscous shear stress and peak maximum shear stress averaged over the RVOT for varying cardiac output levels (bottom left). WSS and peak WSS averaged over the RVOT for varying cardiac output levels (bottom right). All quantities are obtained using 3D-PTV *in vitro*. Red circles correspond to peak values, whereas blue squares represent time averaged values.

This section focuses on the influence of various cardiac output levels on flow characteristics *in vitro* (Table 1). Figure 3 shows the variation of time- and space-averaged MKE, laminar viscous dissipation, maximum viscous shear stresses and WSS with changing cardiac output levels. MKE increases with increasing cardiac output, which is expected as the phase averaged velocities become more intense for a higher cardiac output (Fig. 3, top left). Peak MKE levels also shows the same trend with a steeper increase. Furthermore, it is depicted that laminar viscous dissipation significantly increases by increasing the cardiac output. In particular, a 32% increase in cardiac output leads to an approximately two-fold increase in both MKE and laminar viscous energy loss (Fig. 3, top right). The maximum viscous shear stresses show a similar trend for increasing cardiac output (Fig. 3, bottom left). It is noted that both time and space averaged maximum viscous shear stresses and peak maximum viscous shear stresses increase by 70% for a 32% increase in cardiac output. Finally, a 60% and approximately linear increase in WSS has been found at maximum cardiac output, which is similar to the trends of the time and space averaged maximum viscous shear stresses (Fig. 3, bottom right).

**Table 1:** Model characteristics and flow parameters obtained *in vitro*.

Parameter	Unit	Case I	Case II	Case III	Case IV
Density of working fluid	*kg*/*m*^3^	1200	1200	1200	1200
Dynamic viscosity of blood	*Pas*	5.8 × 10^−3^	5.8 × 10^−3^	5.8 × 10^−3^	5.8 × 10^−3^
End Diastolic Diameter of RVOT	*mm*	19.4	19.4	19.4	19.4
Thickness of RVOT	*mm*	2.5	2.5	2.5	2.5
Volumetric Flux	*ml*/*s*	67.5	73.9	77.5	89.2
Heart Beat	*bpm*	60	150	100	120
Cardiac output	*lt*/*min*	4.05	4.44	4.65	5.32
Peak velocity magnitude	*m*/*s*	0.295 ± 0.02	0.401 ± 0.02	0.413 ± 0.02	0.424 ± 0.02

### The effect of increasing cardiac output levels on *in vitro* WSS

**Figure 4:**
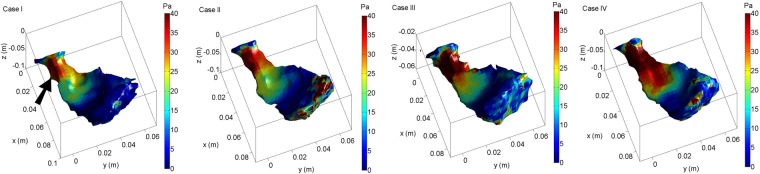
Spatial distribution of WSS at peak systole for varying cardiac output levels obtained using 3D-PTV *in vitro* (Case I: 4.05 L/min, Case II:4.44 L/min, Case III:4.65 L/min, Case IV:5.32 L/min,). Red color corresponds to high WSS, whereas blue color represents low WSS. A substantial increase in WSS in the RVOT, particularly in the anterior RVOT (free wall, black arrow) is observed with increasing cardiac output.

This section shows a comparison of WSS patterns along the RV for varying cardiac outputs. Figure 4 depicts the spatial distribution of WSS at peak systole for different *in vitro* experiments (see Table 1) studied. As seen, highest WSS regions develop at the anterior (free wall) RVOT, followed by the subtricuspid region. Of interest, the WSS in the RV apex and septal region is negligible. Furthermore, in the RVOT WSS linearly increased with higher cardiac output. On the contrary, we did not observe a linear increase in WSS in the subtricuspid region, but WSS in the latter region was highest at maximum heart rate (150 bpm). Furthermore, it is found that the high WSS regions expands towards the RV apical wall with an increase in cardiac output (Fig. 4, Case IV).

**Figure 5:**
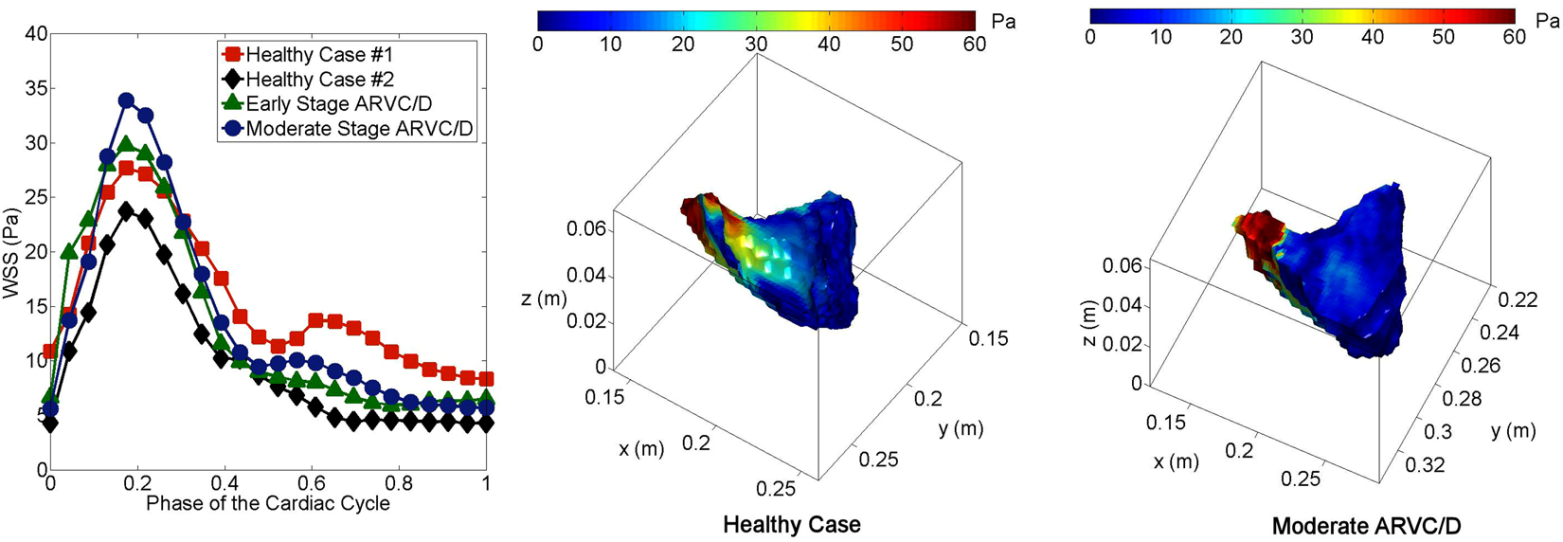
Time evolution of WSS averaged over the RVOT for healthy, early ARVC/D and moderate ARVC/D cases (left) obtained using *in vivo* PC-MRI. Spatial distribution of WSS at peak systole for the healthy case (middle) and moderate ARVC/D case (right).

Finally we compare the behavior of the *in vivo* comparison of WSS between two healthy cases and two ARVC/D cases at resting conditions. As depicted in Fig. 5 (left), peak WSS at the RVOT for the moderate stage ARVC/D case is around 23% higher than that of the healthy cases, while for the early stage ARVC/D case it is 8% higher than the healthy cases, although RVOT dimensions (Table 2) were similar among the three cases. In Fig. 5 (center) and (right), it is shown that the spatial distribution of the WSS along the RV is different for the healthy and diseased ARVC/D case. Higher WSS are found at the RVOT region as compared to the remaining RV for both healthy and diseased cases.

**Table 2:** Subject characteristics and flow parameters obtained *in vivo* at resting conditions. (EDV: End diastolic volume, EDD: End diastolic diameter).

Parameter	Unit	Healthy Case I	Healthy Case II	Early Stage ARVC/D	Moderate Stage ARVC/D
Body Surface	*m* ^2^	1.77	2.01	1.88	1.68
RV Ejection Fraction	%	53	47	42	42
RV EDV	ml	170	204	216	211
EDD of RVOT	*mm*	17	16	17	15
Thickness of RVOT	*mm*	5	5	4.5	4
Heart Beat	*bpm*	66	62	63	65
Stroke Volume	*ml*	90.6	88.2	87.5	91.6
Cardiac output	*lt*/*min*	5.98	5.31	5.51	5.95

## Discussion

In this study, we investigated the effects of various cardiac output levels on RV and RVOT flow patterns under physiological flow conditions *in vitro* and *in vivo*. Fluid mechanics parameters such as MKE, WSS and viscous energy dissipation were utilized to characterize the blood flow along the RVOT. We first presented the blood flow patterns at resting conditions in the right heart of a healthy athletic male volunteer *in vivo* using PC-MRI to highlight the differences between the RVOT and other RV sites, i.e. subtricuspid region, RV apex. Then we presented the influence of varying cardiac output on shear stresses and energy losses *in vitro* using 3D-PTV. To translate our findings into the clinical setting, we compared two healthy cases to two patients with different ARVC/D disease stages.

We found that MKE at the RVOT of a healthy human heart is around one order of magnitude higher than at other RV sites during the systolic phase at resting condition. A similar trend was found in WSS analysis, i.e. high WSS regions develop at the RVOT at peak systole and space and time-averaged WSS at the RVOT is around two times higher than the WSS averaged over the entire RV. This implies that even in the healthy heart at resting conditions RVOT is exposed to high shear stress.

RVOT tachycardia is the most common form of idiopathic ventricular tachycardia, which may occur in the absence of structural heart disease^24,30^. It is suggested that high level endurance exercise training^14,27^ and pregnancy^31^ may provoke idiopathic RVOT tachycardia. Yet, RVOT tachycardia can also occur in patients with ARVC/D^14,32^. A recent study using epicardial bipolar voltage mapping *in vivo* has shown an isolated subepicardial RVOT scar at the RVOT free wall in the athlete’s heart^14^. In our study, we have shown that high WSS regions develop in the RVOT at resting conditions and they intensify in an approximately linear fashion at increased cardiac output levels. Of note, WSS levels were relatively higher at the proximity of the lateral RVOT (free) wall as compared to the septal RVOT and other RV regions for the highest cardiac output investigated. An increase in cardiac output leads to a relative shift of WSS from the septal RVOT to the RVOT free wall, and a relative increase in WSS at the RVOT free wall as compared to the septal RVOT and other RV regions. The identified regions of elevated hemodynamic stresses at resting and exercise conditions correlate with the common site of RVOT ventricular tachycardia in athletes and diseased regions in patients with ARVC/D. Next to RVOT, also the subtricuspid region was associated with elevated WSS. Although WSS did not linearly increase in this region with increasing cardiac output, WSS was hightest at maximum heart rate. These interesting findings may be associated with the clinical observation that the subtricuspid region and the RVOT are the first RV sites to be involved by the pathophysiologic process in ARVC/D^26^. With regard to this, peak systolic velocities were measured in the RVOT, and peak diastolic velocities in the subtricuspid region. Furthermore, our results indicate that even at resting conditions, WSS, disease stage of ARVC/D and ventricular arrhythmia burden as indicated by Holter-ECG monitoring are correlated, i.e. higher WSS regions develop at the RVOT in patients with ARVC/D as compared to the healthy proband.

We also showed that the spatial distribution of the WSS along the RV is different for the healthy and diseased ARVC/D cases although higher WSS zones are located at the RVOT region for both healthy and diseased cases. This finding is interesting as it shows that the hemodynamics of the RV in patients with ARVC/D or the athlete’s heart change as RV remodelling progresses. It is suggested that WSS could be utilized as an indicator for analyzing disease severity in patients with ARVC/D and the athlete’s heart. Based on the results presented in this study, we therefore propose that a relative increase in wall shear stresses in the RVOT, particularly in the RVOT free wall, may contribute to adverse remodelling, scar formation and arrhythmia origin in this region in athletes and patients with ARVC/D.

The pulmonary circulation is a low pressure system, which allows the RV to operate at minimal energy cost^4^. It is well known that large scale structures e.g. diastolic filling vortices exist in the RV^33,34^. However, studying RV hemodynamics in detail has been challenging as a consequence of its complex geometry. In this study, we found that highly vortical regions develop along the lateral wall of the RVOT with increasing cardiac output, and a relative shift of increased WSS occurs from the septal RVOT at baseline to the RVOT free wall at increased cardiac output. These observations may be linked to the physiologic structural adaptations of the athlete’s hearts, i.e. increased volumes^28^, but also pathologic processes such as scar formation in the athlete’s heart and ARVC/D^14,32^.

## Limitations

The pump used in this study has a technical limitation, which limits studying higher cardiac output levels. We were limited to moderate exercise conditions and it was not possible to study intensive exercise conditions with a cardiac output above 20 L/min. It would be worth extending the analysis to this regime in future work since the hemodynamic parameters may become non-linear at a higher cardiac output. Nevertheless, even at a cardiac output of 5.3 L/min equaling a 32% increase from baseline, we observed a substantial and relatively larger increase in wall shear stresses in the RVOT as compared to other RV regions. We can therefore assume that at even higher cardiac output levels such as in endurance athletes, this relative increase would substantially rise further in the RVOT. Therefore, our *in vitro* observations help to understand the susceptibility of the RVOT for structural changes and ventricular arrhythmias in athletes and patients with ARVC/D. Based on this preliminary data and the linear increase of WSS in the RVOT, we cannot make any recommendations regarding a certain cut-off for maximum exercise intensity (e.g. heart rate nor cardiac output) that can be considered safe in patients with ARVC/D. However, we have to acknowledge that the three groups investigated (healthy, mild ARVC, and moderate ARVC/D) have been characterized exclusively from a single individual each, and therefore biological variability has not been taken into account and formal statistical analysis was not possible. In the future, there is a need to reproduce our findings in a larger sample size and the effects of variations in cardiac output on RV hemodynamics *in vivo*.

## Methods

We confirm that all methods were carried out in accordance with relevant guidelines and regulations. This study was conducted in full agreement with the principles of the “Declaration of Helsinki” and current Swiss law. It has been approved by the Ethics Committee of the Canton of Zurich (approval number KEK-ZH-Nr. 2014-0443). All probands signed an informed consent form for prospective inclusion.

### *In vitro* measurements

**Figure 6:**
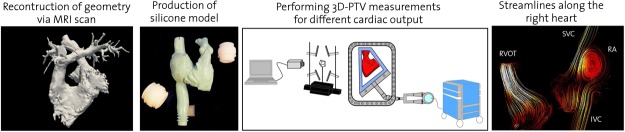
Schematic workflow for the assessment of the velocity information in the novel right heart model. A high resolution magnetic resonance imaging (MRI) heart scan of a healthy male proband has been performed. A silicone model cast was 3D printed out using the geometry obtained via MRI. The right heart model was manufactured by brushing the surface of wax positive (Elastrat GmbH, Switzerland). 3D-PTV measurements have been performed in the anatomically accurate right heart model. Streamlines color coded with velocity magnitude were obtained along the superior vena cava (SVC), inferior vena cava (IVC), right atrium (RA), RV and RVOT.

To investigate the hemodynamic flow patterns in the right heart *in vitro*, an optical imaging tool, 3D-PTV, has been applied to an anatomically accurate compliant silicone phantom (Elastrat GmbH, Switzerland) of the human RV and RA derived from a high resolution MRI heart scan of a healthy male proband. The experimental setup, which is similar to the one used in the study of Gülan *et al*.^22^, comprises a physiological silicone model, a high speed camera (Photron SA5, Japan), a diode-pumped Nd-YLF laser (Quantronix, Darwin Duo 527 nm, USA), an image splitter, a data acquisition system, and a wave generator (Fig. 6, center).

The material of the right heart model is a permanently cured, but flexible silicone from DOW Chemicals. The model is manufactured by brushing the surface of wax cast and curing the layer to the elastic state^35^. The heart model allows active expansion and contraction of the RV using a custom designed pressure and vacuum pump. In the atrial filling and ventricular systolic phase, the RV contracts and forwards the fluid flow towards the pulmonary circulation, whereas the atrium expands as a consequence of the inflows from both venae cavae. In the ventricular filling phase, the RV expands as the contraction of atrium forwards the flow towards the RV. The chamber volumes of this model are similar to those in clinical studies^28^.

We performed our experiments at four different cardiac output levels, namely 4.05 *L*/*min*, 4.44 *L*/*min*, 4.65 *L*/*min*, and 5.32 *L*/*min*. The flow recordings were performed with full resolution of 1024 × 1024 pixels and 2000 frames per second with a Nikkon AF Micro Nikkor 60 *mm* f/2.8 D lens (Japan). The high-speed camera was synchronized with the heart pump system to trigger the recordings at the beginning of every cardiac pulse. A 100 W laser power was used for illuminating the fluorescent rhodamine particles (Cospheric, USA) with a diameter of 200 *μm*. A mixture of glycerine, water and sodium chloride was used as working fluid, which matches the refractive index of silicone (n = 1.41) and the kinematic viscosity of blood (4.85 × 10^−6^*m*^2^/*s*)^36^. The position accuracy of the particles was 0.17, 0.17 and 0.35 *mm* in x, y and z directions, respectively for all cardiac output measurements. The velocity uncertainty of the raw PTV data, as obtained from the calibration, was 0.038 *m*/*s*. The Lagrangian data points were indexed onto a homogenous Eulerian grid system with a voxel size of 3.2 × 3.2 × 3.2 *mm*^3^. A total of 40 cycles were recorded to obtain a phase averaged flow field which corresponds to 120 k images allowing obtaining converged statistics for both phase averaged flow fields and velocity fluctuations. The heart rate was varied from 60 *bpm* to 150 *bpm*. A summary of conditions studied in this study is shown in Table 1.

### *In vivo* measurements

*In vivo* 4D-PC MRI measurements were performed with a 3 T Philips Ingenia System (Philips Healthcare, The Netherlands) to extract the physiological phase averaged velocity field in two healthy athletic male probands’ RV, and 2 additional patients with different stages of ARVC/D, namely early and moderate stages, which were assigned by RV dimensions and ventricular arrhythmia burden as assessed by ECG/Holter-ECG. The imaging parameters were as follows: spatial resolution 2.5 × 2.5 × 2.5 *mm*^3^, field of view 250 × 160 × 50 *mm*^3^, TR/TE 5,8/3,4 ms, and temporal resolution 35 ms. The velocity encoding (VENC) values are 40, 100 and 200 cm/s per direction. The phase contrast sequences were synchronized with the electrocardiography (ECG). A 3D-gradient echo phase contrast sequence with multi-point velocity encoding was applied to obtain 10x k-t undersampled data^37^. Subject characteristics and flow parameters obtained *in vivo* are summarized in Table 2.

### Mean kinetic energy analysis

Mean kinetic energy (K), which is the square of the phase averaged velocity magnitude, is defined as

1 $$K=1/2({U}_{i}\mathrm{.}{U}_{i}),$$

where *U*_*i*_ is the phase averaged velocity vector.

### Maximum viscous shear stress analysis

Viscous shear stresses are defined as

2 $$VSS=\mu (\frac{\delta {U}_{i}}{\delta {x}_{j}}+\frac{\delta {U}_{j}}{\delta {x}_{i}}),$$

where *μ* is the dynamic viscosity of the fluid. The maximum viscous shear stress is calculated as

3 $$VS{S}_{max}=2\mu ({e}_{1}-{e}_{2}),$$

where *e*_1_ and *e*_2_ are the eigenvalues of the stress tensor.

### Laminar viscous dissipation

The viscous dissipation of MKE due to mean shear, i.e. gradients of the mean velocity profile is defined as,

4 $$\varepsilon =2\nu {S}_{ij}{S}_{ij},$$

where *ν* is the kinematic viscosity of the fluid and ${S}_{ij}=1/2(\frac{\delta {U}_{i}}{\delta {x}_{j}}+\frac{\delta {U}_{j}}{\delta {x}_{i}})$ is the mean strain rate tensor.

### Wall shear stress analysis

The viscous WSS at a certain time instant is a tensor calculated as

5 $$\tau =\mu ({S}_{ij}),$$

We use the L2 norm of the WSS tensors to quantify WSS magnitudes. The wall shear stresses are calculated using a velocity interpolation to positions with fixed normal distance to the detected boundary^22^. We achieve a resolution of O(1 mm) in our measurements and use a cubic spline to interpolate the wall-normal velocity profile and estimate its gradient.

### Strain analysis

To further compare *in vivo* and *in vitro* conditions, strain measurements were performed both in the silicone phantom and in the *in vivo* heart. 3D-PTV data was used to extract strain in the silicone phantom. The strain is calculated as

6 $$\gamma =(L-{L}_{0})/{L}_{0},$$

where *L*_0_ is the initial length of interest and *L* is the length at a certain time instance.

*In vivo* strain measurements were performed using 2D transthoracic echocardiography from the apical four-chamber view at rest (Vivid 9 echocardiography scanner, GE Medical Systems, Horten, Norway). Deformation imaging by speckle tracking is a technique, which is an angle-independent method avoiding limitations related to translational cardiac motion. The endocardial border was traced and the region of interest was adjusted to the myocardial wall. Wall motion was tracked over the cardiac cycle. Myocardial speckle tracking was digitally analyzed using Echo Pac Work Station (GE Medical Systems).

#### Comparison of *in vitro* and *in vivo* RVOT flow patterns

In an *in vitro* model, we investigated the influence of various cardiac output levels on RV hemodynamics. RVOT flow patterns at resting conditions were obtained in a athletic healthy proband’s heart (healthy case #1) via PC-MRI, which is used as a reference for validating the *in vitro* flow patterns.

**Figure 7:**
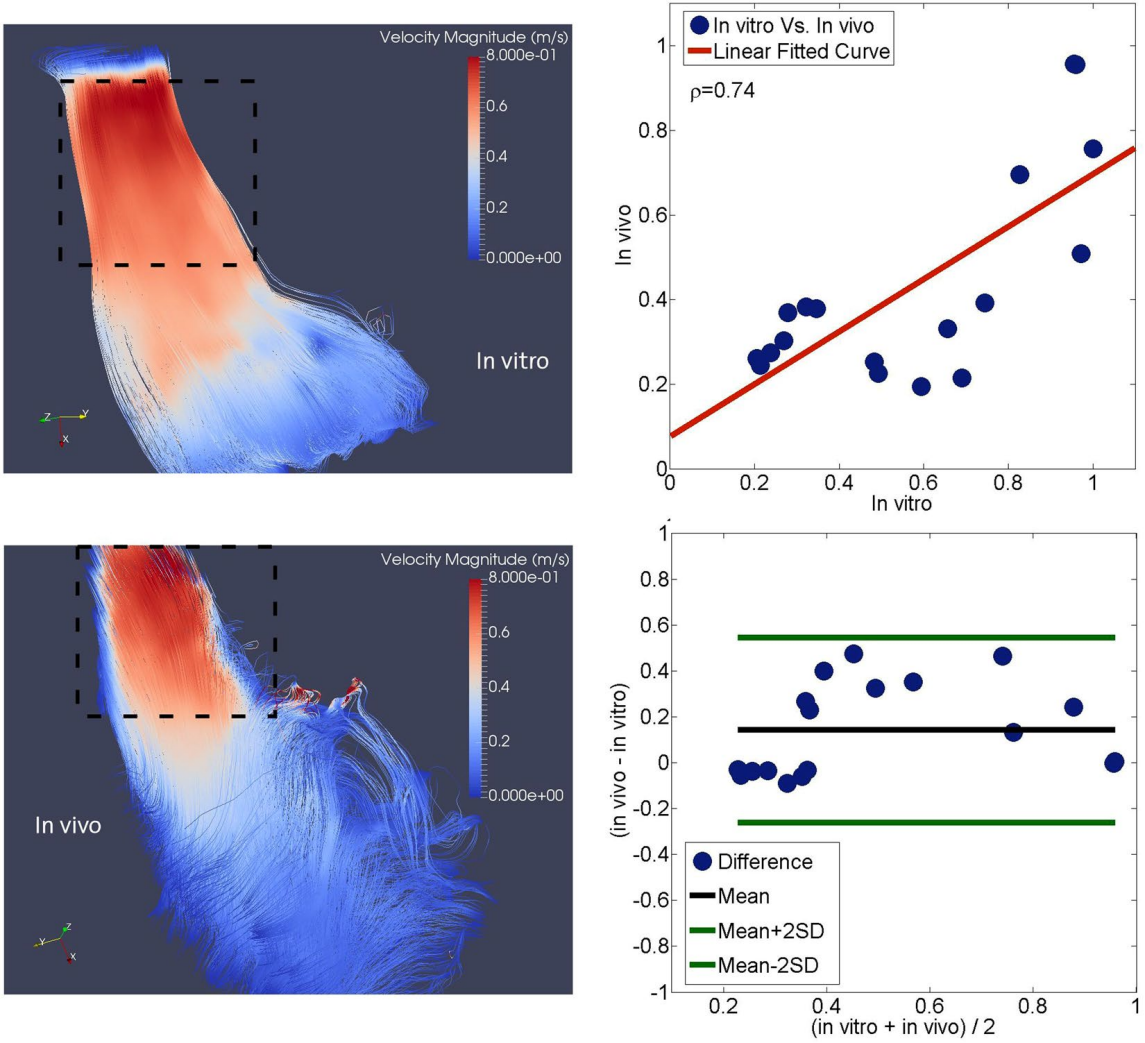
Comparative analysis of the *in vivo* and *in vitro* flow fields. Streamlines color coded with velocity magnitudes at the peak systolic phase *in vitro* (top left) and *in vivo* (bottom left) (higher velocity magnitudes occur in the proximity of the RVOT, whereas the velocity magnitudes are comparatively lower at the RV subtricuspid region and the apex). Dashed lines represent the investigation domain where the statistical analysis has been performed. The linear regression analysis between velocity magnitude normalized by peak velocity magnitude *in vivo* and *in vitro* (top right) and Bland-Altman analysis between velocity magnitude normalized by peak velocity magnitude *in vivo* and *in vitro* (bottom right). Filled blue circles represent a different time-point in the cardiac cycle.

The boundaries of the RVOT and RV were defined according to clinical practice (cardiac MRI). Figure 7 depicts both a qualitative (left) and quantitative (right) comparison between *in vivo* and *in vitro* (Case IV) measurements. There is qualitatively good agreement in the RV flow patterns at the peak systolic phase. High velocity zones occur in the proximity of the RVOT, whereas the velocity magnitudes are comparatively lower at the RV subtricuspid region and the RV apex (Fig. 7, left). Statistical analysis of velocity averaged over the RVOT region shows that there is a good agreement in peak velocity magnitudes between *in vivo* and *in vitro* (Fig. 7, right; correlation coefficient *ρ* = 0.73). Phase averaged flow parameters obtained both *in vivo* and *in vitro* are summarized in Table 3. Overall, there is a good agreement in the RVOT velocity magnitude, MKE, vorticity magnitude and associated stresses between the model and the *in vivo* results. There is a slight difference in peak systolic values.

**Table 3:** Comparison of the flow parameters obtained both *in vivo* and *in vitro* (Case IV).

Parameter	Unit	*In Vivo* (Healthy Case #1)	*In Vitro*
Peak RVOT velocity magnitude	*m*/*s*	0.451 ± 0.04	0.424 ± 0.02
Mean RVOT velocity magnitude	*m*/*s*	0.194 ± 0.03	0.241 ± 0.02
Peak vorticity	1/*s*	50.78 ± 4.5	61.99 ± 3.7
Mean vorticity	1/*s*	33.07 ± 4.5	36.32 ± 3.7
Peak VSS	*J*/*m*^3^	0.396 ± 0.02	0.376 ± 0.02
Mean VSS	*J*/*m*^3^	0.257 ± 0.02	0.226 ± 0.02
Peak WSS	*Pa*	30.52 ± 3.5	39.48 ± 2.6
Mean WSS	*Pa*	17.01 ± 3.5	21.35 ± 2.6

#### Comparison of *in vitro* and *in vivo* RV contraction

**Figure 8:**
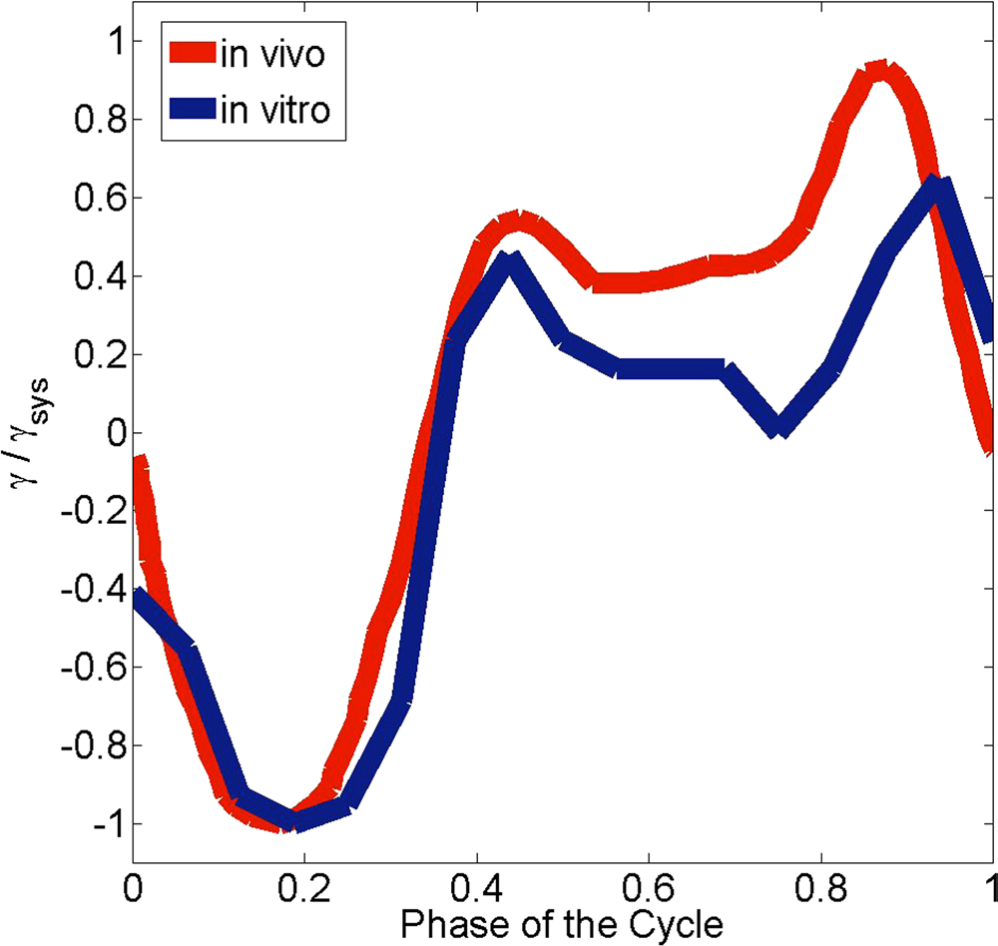
Time evolution of the *in vivo* longitudinal strain normalized by peak systolic strain at RV free wall and *in vitro* strain normalized by peak systolic strain at the RV free wall (red color represents *in vivo* strain and the blue color represents *in vitro* strain).

The physiological RV contraction in human begins with activation in the septum, extending to the RV free wall. To validate the presented model may produce similar hemodynamics to the *in vivo* conditions, time evolution of the *in vivo* and *in vitro* longitudinal strain normalized by peak systolic strain along the RV free wall is shown in Fig. 8.

There is a good qualitative agreement in the temporal variation of the longitudinal strain between *in vitro* and *in vivo* measurements, i.e. the temporal trend of the strain shows a negative kink during systole and two peaks in the diastole. In the early systolic phase the strain is negative representing the contraction of the RV, while pressure in the RV increases. At peak systole the strain reaches the maximum magnitude. In the deceleration phase the RV relaxes and the pressure decreases. In the early diastolic phase, pulmonary valve closes and tricuspid valve opens leading to a further decrease in pressure and an increase in positive strain, i.e. the RV starts expanding as the fluid flows from the right atrium towards the RV. During the isovolumetric contraction phase, the RV again starts to contract and the cardiac cycle restarts. The agreement between *in vitro* and *in vitro* strain is better during the systolic phase, whereas some discrepancies arise during the diastolic phase.
